# Evaluating Age-Related Variations of Gaze Behavior for a Novel Digitized-Digit Symbol Substitution Test

**DOI:** 10.16910/jemr.12.1.5

**Published:** 2019-06-20

**Authors:** Debatri Chatterjee, Rahul Dasharath Gavas, Kingshuk Chakravarty, Aniruddha Sinha, Uttama Lahiri

**Affiliations:** Embedded Systems and Robotics, TCS Research and Innovation, Kolkata, India; Centre for Cognitive Science, IIT Gandhinagar, India

## Abstract

Analysis of cognitive functioning from gaze behavior might serve as an early indicator of age related decline of cognitive functions. Standard psychological tests like the digit-symbol substitution test or the symbol-digit modalities test is used exclusively in this regard. In this paper, we have designed and developed a digitized version of the digit symbol substitution test. Three different versions have been designed in order to derive deeper insights of the user behavior. The test-retest validation of the versions reveals good correlation across sessions. Further, the difference in gaze behavior which might be used as an indicator of cognitive functions is tested for two different age groups (13 participants <30 years and 11 participants >40 years). It is seen that the designed digitized version along with the usage of physiological markers like eye tracking bestows additional information and is sensitive to age related factors which might be used for the assessment as well as for the training purpose in rehabilitation systems. Results show that the performance can be analyzed using gaze and pupillometric features in addition to the conventional test performance metrics. We derived an index to measure the performance related to visuo-spatial functioning on one of the designed versions of the test. Results of this index on the number of fixations for two age groups are found to be separated in a statistically significant (p<0.05) manner. The age related difference (p<0.05) is also evident in the pupillometric responses obtained.

## Introduction

Digit Symbol association test (DSST) is one of the test batteries to study cognitive functions [1]. DSST is indicative of various factors like processing speed, visual scanning, motor response, cognitive processing and working memory [1]. The task involves the harnessing of fluid cognition, thereby making it a putative marker for studying cognitive functions, age related variations in cognitive performances and decline [2]. The test performance in this regard correlates strongly with cerebral atrophy [3], brain lesion volume [4], retinal nerve fibre layer thickness [5] and diffusion tensor indices of brain tissues that might appear normal otherwise [6]. Recent studies show that in conjunction with other standard cognitive tests, DSST reflects the qualitative nature of the self-reported cognitive impairments [7].

**Figure 1.**
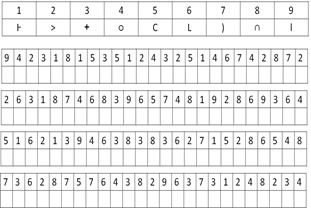
An illustration of the Pen and paper DSST (pDSST)

In the present study, we have used the pen and paper version of the DSST (pDSST) from National Institute of Mental Health and Neurosciences, NIMHANS, Bengaluru [8] and proposed a modified digital version of it. The pDSST requires the association of digits with symbols with reference to a lookup table (Fig. 1). There is a lookup table consisting of nine numbers with nine associated symbols followed by a sequence of numbers with blank boxes. Participants are required to fill the matching symbols as per the lookup table entries. The figure is only for illustration purposes, so that the readers get an idea of the test and by no means do the number-symbol pairs in the lookup area and the following sequence of numbers in the entries of the figure depict the pairs used for actual data collection. The task performance is assessed based on the number of correctly associated symbols. Also, the total time taken to associate all the given digits with their corresponding symbols is used as a performance metric. Recently, attempts have been made to digitize this test. These computer-based tests allow automation of scoring, renders dynamic manipulation of the components in the task, and are sensitive to age [9] as well as the effects imposed by drugs [10]. These findings motivated us to study if we can get a deeper insight about the cognitive functionalities of healthy individuals with the help of this test.

In order to get additional information about the cognitive performance of an individual, we have used a low cost eye tracker to get direct insights on the gaze pattern of the participants while they are trying to match a number with its associated symbol. As this matching step is at the core of the DSST, eye tracking is a good means of analyzing the behavior of the participant. The usage of gaze tracking is crucial to understand the implications of paired associations made during recall. Completion of a trial (matching a digit to its symbol) without the usage of the lookup table is indicative of the use of learned/memorized paired associations. Gaze analysis reveals that few participants tend to memorize the lookup table more [11] compared to others. Hence we have designed a version where the lookup table entries (i.e. digit-symbol pair) change with each trial. This would give rich information elucidating the importance of paired association.

There are various reasons that make it necessary to have the digitized version of the DSST task which are mainly associated with the shortcomings of the conventional pen and paper DSST test. Firstly, in the pDSST, the assessment is purely based on the correct matches done within the given time interval. It does not consider the gradual changes occurring in the response time, attention, working memory and visuo-motor coordination across trials. Also, the existing approaches focus mainly on the results pertaining to the entire task duration. However, with the digitized approach, the analysis and correlation of the test results, user responses and physiological changes across the trials is possible. Though there are number of existing digitized versions of the test [12, 13, 14], unlike the currently existing ones, our approach provides flexibility to the designer to easily modify the design for assessing various mental states of an individual.

The objective of the present study is to validate a newly designed computer based digitized DSST (dDSST) and to extract information about cognitive performance of the participants under test. In order to do so, the test-retest validation of the designed version is done in comparison to the conventional pen-paper DSST (pDSST). In addition to this, we have analyzed the age related effects on the cognitive performance. The usage of gaze tracking coupled with pupil response has helped us to study the variations occurring in the processing speeds due to age.

The paper is organized as follows. The currently existing systems have been discussed in “Related work” section followed by study methodologies in “Methodology” section. “Experimental Paradigm” details the experimental setup, protocols and participants selected for the study. Results have been discussed in “Results and Discussions” section. Finally the “Conclusions” section summarizes our observations along with scope of future work.

## Related Work

One of the earlier attempts in digitizing the Symbol-Digit modalities test (SDMT) was done by Daniel et al [15]. In that test, the user had to reproduce the test query symbol on a numeric keypad and 25 sessions, of 90 seconds each were conducted. It was seen that the performance scores increased with each session. Response to the stimulus demanded more of motor abilities since reproducing each symbol required pressing three buttons on the keypad (per trial). This feature hampered the classification of impairments related to processing speeds when the target subjects were Schizophrenic or had other cognitive impairments or limited motor abilities. Again, most of the existing digitized versions of DSST feature a fixed duration for each trial. For example, in one of the previous attempts to digitize the SDMT task, Forn et al [16], designed a version in which the stimulus appeared for a fixed duration of 2 seconds in order to make it compatible with the protocol of fMRI data collection. There exist both advantages and disadvantages of this approach. Specifically, the fixed duration of 2 seconds can be used to detect the inadvertently imposed anxiety. In contrast, such a short duration might not be sufficient to detect the complete underlying processes involved while performing a task.

In SDMT the positions of digits and symbols are reversed with respect to DSST. In case of SDMT, the digits are matched against the symbols and digits are to be written down or spoken out, unlike DSST wherein symbols are to be identified and written down. SDMT is comparatively harder than the DSST and is more sensitive to neurotoxicity, although the performance of both SDMT and DSST is observed to be highly correlated in the works of Lezak [17].

Since the pen and paper version demands the psychomotor abilities, Mcpherson et al [18] came up with a digitized SDMT in which responses are collected through mouse click events. In [19], the authors came up with a gamified version of the digitized SDMT that correlated well with processing speed tasks like the Digit-Symbol Visual matching, decision Speed tests and with working memory tests like “picture Swaps” and “Dot Matrix tests”. The digitized version, though powerful in identifying the differences in performances across participants based on their explicit moves, needs more input to identify the underlying reason behind such differences [20]. One of the avenues to achieve this is through use of implicit measures such as physiological changes that are often not affected by motor impairments.

The inclusion of physiological sensing is difficult in case of pDSST, however, Elahipanah et al [21] used a projected version of pen and paper SDMT on a computer screen to incorporate eye tracking and there the participant was supposed to read out the numbers corresponding to the query symbols. The eye movement behavior was analyzed and considerable variances with respect to fixation and saccades on the stimulus area were noticed. Though a computerized version is expected to provide the details for each entry, it is difficult to do that just by putting the entire DSST page on a single screen. Hence, there is a need for complete digitization of the task. This would allow us to easily configure font size, number of trials, change the difficulty levels or introduce any new feature in the test. The work of Akbar et al [14] has used verbal response, however, in that they have come up with the digitized version of SDMT wherein 8 query symbols were shown in a trial. Elahipanah et al [21] evaluated the eye movement behavior and considerable variances with respect to fixation and saccades on the stimulus area were noticed.

Apart from the eye gaze tracking, researchers have been investigating on the use of other modalities such as verbal response, key stroke, etc. For example, the work of Akbar et al. [14] has considered one’s verbal response while the digitized version of SDMT having 8 query symbols per trial was used. Again Bachman et al [22] digitized the SDMT and the response was taken mainly based on two key strokes- one for correct match and the other for incorrect match for the query appearing in each trial. The performance was distinguishable for both healthy controls and the ones suffering from Schizophrenia. The study was further extended by Amaresha et al [13] wherein the restriction in relational memory was shown as an imperative factor for reduced processing speed. All the existing literature mainly relies on the overall task performance and accuracy as is done in pen and paper versions.

Among the different modalities of picking up one’s response to a task, eye tracking is one of the promising modalities in the domain of cognitive performance analysis. Gaze analysis along with pupil response provides valuable insights of the cognitive workload [23], confidence [24] and decision making [25] abilities of an individual. Nicolas et al [26] had shown the possibility of emotion analysis using fixation duration of the eye for depressed older adults and normal controls. Van der et al showed that various features of eye movements (e.g location of fixation, correction in gaze, and so on) are largely governed by the visuo-spatial working memory [27]. The representations in working memory are indispensable parts of target selection, correcting the gaze when the eyes fail to land on the target entity, and so on [27]. Given the potential of gaze information to be closely linked with the underlying cognitive processes, in our present study, we have used the gaze data to get insights into the age-related manifestations (in terms of looking pattern) among the participants when exposed to a digitized version of DSST.

## Methodology

### Motivation of the proposed study

Researchers have been exploring the use of digitized version of the pen and paper format of DSST that can offer a number of benefits. Specifically, from the designer’s perspective, the digital version would allow one to easily configure the font size, number of trials, change the difficulty levels or introduce any new attribute to the test. As regards the experimenter, the digital version can record the time taken by a user during each trial quite accurately. Again, digitization enables the DSST platform to employ automated scoring mechanisms. Additionally, it enables the platform to be seamlessly integrated with external peripheral devices such as physiological sensing. Unlike considering the test performance score in isolation, the physiological sensing coupled with performance scores can potentially provide stronger insights into the underlying cognitive processes. Thus, this can serve as complementary tool in the hands of skilled professionals in the field.

### Design Philosophy

For designing the digital version of DSST, we have borrowed concepts from the standard pen and paper version of the DSST. Since we wanted to study one’s gaze behavior added to the task performance, our design took into account the incorporation of the eye tracker.

**Figure 2.**
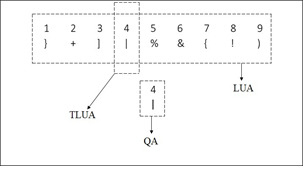
Proposed digitization scheme of DSST

A schematic layout of the proposed digitized DSST test is shown in Fig. 2. The layout includes the following: A look up area (LUA) fixed at the top which contains the target symbol-digit pairs, and the query symbol-digit pair appearing at a predefined position on the screen, called query area (QA). The region of LUA having same digit as QA is termed as the target LUA (TLUA) as shown in Fig. 2. If the query symbol-digit pair matches with the entries in the look-up table, the user presses the *space bar*. For every trial, the participant has to do the following, check the digit-symbol pair presented in the query area, search for the same digit in the lookup region, match the digit-symbol pairs of TLUA and QA and respond accordingly.

For each trial, the user needs to respond within a pre-determined duration (we have chosen 3 seconds). Please note that we have chosen the duration of 3 seconds as a typical value based on the feedback of participants from an initial pilot study. This can be easily modified based on the task paradigm. As far as user’s response is considered, for a non-matching digit-symbol pair (between that in TLUA and QA), the user’s response was correct if the user did not press any button and instead waited for the next query pair. Vice-versa was the case for a matching pair. For every correct response, the performance score was incremented. The duration of each trial was selected based on the feedback of the participants from an initial pilot study. In the current study, all the participants agreed upon 3 seconds as the default duration. The inter-trial delay (between the user response of one trial and the display of the content for the next trial) is also selected through user feedback and is taken as 300 milliseconds. The total number of trials is chosen to be 50 with each trial lasting for 3 seconds. Hence, the maximum time of each session is 50*3=150 seconds. However, out of the 50 trials, 25 trials are with correct matches (where user response is expected) and 25 incorrect matches (where no user response is expected). The total time for a session depends upon the response time of the participants. For instance, if the average time taken by the participant to respond to the correct match is approximately 1 second, then the expected time to complete the task should be (25*3)+(25*1) = 100 seconds. This duration is comparable to that of the earlier attempts of digitized version of symbol-digit matching task [15], which is 90 seconds. Hence the choice of 50 trials for the present experiment is justified. However, these values are configurable and can be changed based on the feedback from the participants. The test can be adapted to any regional language. It is to be noted that the user’s response in the designed version is through a single button press from the user. In our experiment we have used the space bar as it is the largest button on the keyboard. This is done in order to reduce the anxiety or the confusion levels occurring as a result of handling multiple inputs. The proposed digitized DSST test has been designed with three different versions with increasing level of difficulty or the demand for working memory as explained below.

#### Version 1

This version is similar to the conventional pen and paper DSST. For this version the entries appearing at LUA are fixed for each trial and the query symbol-digit appears at the center of the screen (QA). Task parameters like the response time per trial, total time and performance score along with physiological indices as potential indicators of one’s mental state were recorded. This version was used to understand one’s working memory.

#### Version 2

This version is similar to the version 1 except that the digit-symbol pair in the LUA changes pseudo-randomly with each trial. The randomness was used so as to overcome any possibility of participants memorizing the entries of LUA (that were fixed in the case of Version 1).

#### Version 3

This version aims at assessing the performance when the location of the query area changes with each of the trials and the user is expected to have better spatial visuo-motor coordination to complete the task. It also intends to assess the effects pertaining to the positional changes of the query area that might be indicative of one’s visual neglect.

Based on the user’s response and the type of match per trial (correct/incorrect), we segregated the trials into four classes as shown in Table 1. The ‘0’ (or “1”) in the first row denote that the digit-symbol pair on the QA doesn’t (or does) match with that in the LUA. The ‘0’ (or “1”) in the row of *Response* denote that the user didn’t (or did) respond in the trial. Classes A and D correspond to correct responses while the classes B and C are variants of incorrect responses. A summary of the explanation of each of the versions of dDSST and pDSST along with their significance is given in Table 2. Class D sample is of more interest to us because it represented the correct match of digit-symbol pair in QA and LUA. Here, the participants also give his/her correct response. Hence, this set of samples is used exclusively for further analysis.

**Table 1:** Segregation of the trials into classes

**Class**	**A**	**B**	**C**	**D**
**Match**	0	0	1	1
**Response**	0	1	0	1

**Table 2:** Comparison of the different versions of DSST

**Test type**	**Explanation of test**	**Significance**
pDSST	1) 100 entries. 2) User enters symbols manually.	A standard test [8] used for monitoring working memory, visual-motor coordination and attention.
dDSST v1	1) 50 trials. 2) The lookup table entries are fixed for each trial 3) The query pair appears at the center of the screen.	User matches pairs shown in QA with that presented in the LUA. After few trials, the gaze transitions are expected to be less due to the memorizing effect. Lesser the number of transitions, better is the memory.
dDSST v2	1) 50 trials. 2) The lookup table entries change with each trials 3) the query pair appears at the center of the screen.	As the entries in the LUA changes every time, the user needs to check the LUA in every trial. Slower the transitions, lower is the processing speed.
dDSST v3	1) 50 trials. 2) The lookup table entries are fixed and the query pair appears at different locations on the screen.	The user needs to look at different locations of the screen owing to the QA area. This version is indicative of the visuo-spatial functioning. This might also help in detecting left and right visual neglect.

The features used for the study is given in equation 1,

**(1):**
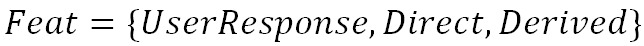


The *UserResponse* corresponds to the performance indices of the test like the task completion time or the response time or the total performance score.

The *Direct* and the *Derived* features are the ones extracted from the eye tracking data. The *Direct* features are fixation durations or the number of fixations in LUA, QA and TLUA along with the pupillary dilation. To get deeper insights of the behavioral patterns as predicted from the gaze data, we proposed extracting the *Derived* features e.g., scanning index and the performance index using the scanpath of the gaze data.

### Eye Tracker Data Analysis

**Figure 3.**
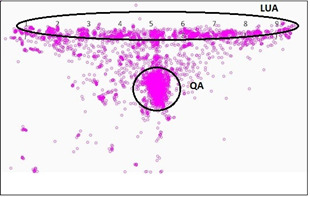
Sample gaze map on DSST

Eye tracker records raw eye gaze data ( *x*, *y*) consisting of fixations and saccades. Fixations are the instances when the gaze is nearly static and information is retrieved from the region of interest. For extracting fixations from the raw eye tracker data, we have used the velocity threshold-based method [28] with the threshold value of 20 pixels/second. The data points lying above the threshold velocity are treated as saccades and the rest are categorized as fixations. Fig. 3 shows a sample gaze map for one particular participant over 50 trials. This helped us to extract the *Direct* features from the gaze data. These features are the fixation durations in LUA, QA and TLUA, the number of fixations while one fixated on LUA, QA and TLUA. The pupillary responses are also analyzed. The proposed *Derived* features from the *Direct* features are the scanning index and the performance index discussed in the following section:

For analyzing the scan path, the transition from QA and LUA is analyzed. Ideally, the transition sequence should be “QA→LUA→Response” or “QA→LUA→QA→ Response”. If a participant is looking at the non-target LUA or other non-region of interest regions of the visual stimulus screen (beyond the targeted regions of interest), then this might indicate that the participant is having attention-related problems. Table 3 summarizes the inferences that can be made about the gaze behavior based on one’s fixation and scan path during a trial.

**Table 3:** Possible findings from eye tracker data

**User-specific inferences based on gaze behavior**	**Gaze behavior based on the analysis of Eye Tracker data**
User is not attentive	Fixations in non-target LUA or no fixations in TLUA immediately after looking at QA
User not sure about the task to be performed	No fixation on TLUA
User has poor memorizing effect	Multiple transitions between QA and LUA
Visual neglect	For instance, consistent wrong answers for query pairs appearing in left or right visual field in version 3
Less processing speed	Transition speed for QA to LUA is low

We have derived a metric called Scanning index (*S*) to capture the information related to scan path. The metric is defined as,

**(2):**
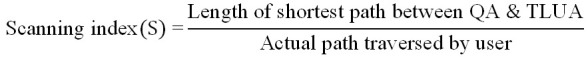


As mentioned before, we considered only class D data (Table 1) while deriving one’s scan path. This was followed by a density-based clustering method [29] applied on the fixation coordinates recorded by the eye tracker data. Also, we considered the corresponding time information while extracting the data on fixation to compute the sequence of the actual path (scan path) traversed. Clusters having density less than a threshold of 90% of the total data in the trial were rejected. The threshold level was derived empirically considering the morphology of the fixation data. Finally, the center of these clusters was connected and curve fitting (2D spline fit) was carried out for data smoothening. Then the Euclidean distance between the QA and the TLUA considered to be the shortest path between QA and TLUA is computed. Ideally *S* should be 1. The value decreases with increase in processing time of an individual. If *S*>1 then the actual path traversed is less than the shortest path between QA and TLUA. It signifies that the participant is not looking at the TLUA. For version 1, if the participant’s response is correct and *S*> 1 then it might imply that the participant is able to memorize the entries of the lookup table and hence, the participant is not looking at it for each trial. Similarly, if the response is wrong and *S*> 1 for version 1 or *S*> 1 for version 2, it might indicate that the participant is not attentive enough while doing the task.

**Figure 4.**
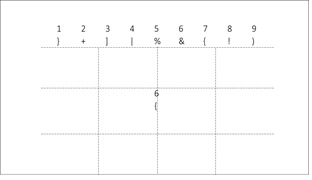
Division of QA for evaluation of performance index in version 3

For version 3, we derived a metric called Performance index ( *H*) for analyzing the performance of an individual for QA appearing at various screen locations. The screen area where the query pair might occur has been divided into *M* number of rows and *N* number of columns as shown in Fig. 4. Thus any query location on the screen can be represented by any one of *M* × *N* rectangular bins. In our case, we have taken *M* = 3 and *N* = 4. For computation of the Performance index, any of the Direct or Derived features could be used.

Next, we compute the probabilities (*p_ij_*) of the query pair to appear in each of the bins with the average feature values using normalization such that the sum of all the *M* × *N* entries is 1, where 1<=*i*<=*M* and 1<=*j*<=*N*. Once probability values are found out, the performance index metric is computed as,

**(3):**
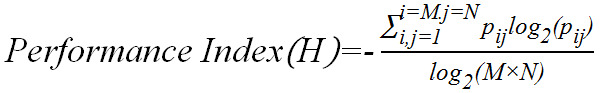


The maximum value of *H* can be 1 when all the bins are equally probable, indicating best performance. The minimum value of *H* can be 0 when any one bin has probability of 1, indicating worst performance. It is to be noted that *H* is computed on the *UserResponse* like the response time and on the *Direct* features (like number of fixations in LUA, QA and pupil dilation) explained in equation 1.

## Experimental Paradigm

### Experimental Setup

**Figure 5.**
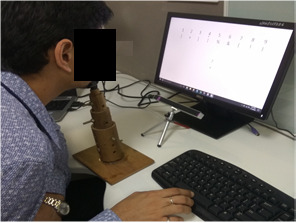
Experimental setup

The stimulus is shown on a 17 inch computer screen (1366 × 768 resolution) placed at a distance of approximately 60 cm from the participant. We have used a chinrest fixed on the table in order to ensure minimal head movements and accurate gaze tracking. The experimental setup is shown in Fig. 5. A low cost infrared eye tracker from Eye Tribe [30] is used to collect the gaze and the pupillometric data at a sampling rate of 30 Hz during the experimental session. This sensor is placed below the computer screen. The test is conducted in a quiet and closed room under constant lighting conditions in order to avoid external distractions to the participant.

### Participants

All the participants are recruited from our research lab having similar educational and cultural backgrounds. They have normal or corrected to normal vision with spectacles.

In our present research, we performed three types of experiments namely, (i) Experiment 1: validation of the dDSST version 1 by comparing against the existing pDSST, (ii) Experiment 2: test-retest validation of the three versions of dDSST and (iii) Experiment 3: analysis and evaluation of the age related differences in the user response, eye gaze behavior and pupillary characteristics.

For Experiment 1, we recruited 14 participants (eight females, mean age ± SD: 28 ± 6 years).

For Experiment 2, a separate group of five participants (2 females, mean age ± SD: 32 ± 8 years) were recruited for experiment 2.

For experiment 3, we selected 13 new participants (four females) with age below 30 years and 11 (three females) relatively elder participants with age above 40 years. The younger age group category will hereafter be termed as *C1* group and relatively aged category as *C2* group. Participants from both the age groups are devoid of any pre-existing cognitive impairments and have normal visual abilities. We selected the age cutoffs for the two groups *C1* and *C2* based on the following analysis. We first subdivide the participants into the following groups as shown in Table 4.

**Table 4:** Subdivision of participants in each group

**Group**	**Age (in years)**
*C1*, Sub-Group 1	<=25
*C1*, Sub-Group 2	>25 and <=30
	
*C2*, Sub-Group 1	>=40 and <=45
*C2*, Sub-Group 2	>45 and <=50
*C2*, Sub-Group 3	>=50

We use the response time as a feature to analyze the within-group differences to justify the division of participants in the categories of *C1* and *C2*. The results of response time for the corresponding Class D samples in each of the 3 versions are compared in the following figures 7 through 8.

**Figure 6.**
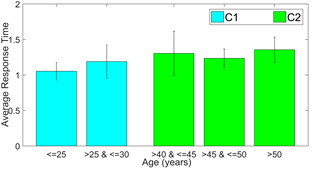
Average response time for dDSST version 1

**Figure 7.**
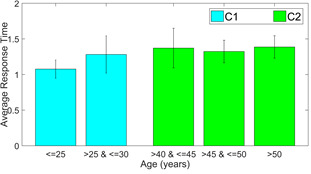
Average response time for dDSST version 2

**Figure 8.**
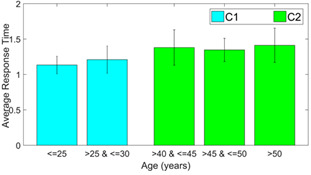
Average response time for dDSST version 3

It is to be noticed that for all three versions, there is very less difference between response times of participants of age below 25 years and between 25 to 30 years. On the other hand, response time changes considerably for the participants with age above 40 years. The inter-group response time is statistically different (p<0.05, effect size = -0.4494, -0.5322 and -0.5677, respectively for the 3 versions of dDSST). The error bar in the plots gives the standard deviation in each case. This justifies the selection of the age range cutoffs during the creation of classes C1 and C2.

### Data Collection

Once the participant arrived at the study room, he was asked to sit down on a chair. The experimenter described the task that the participant was expected to carry out and the study protocol. A demo version of the task is presented before starting the actual data capture in order to ensure that the participants understood the tasks properly. The participants sign a consent form confirming that they participated in the study willingly. The clearance on ethical issues for handling and analysis of the data collected has been acquired from Institutional Review Board of Tata Consultancy Services Ltd. (TCS). The participants are asked to sit comfortably on a height-adjustable chair and place his / her chin on a chin rest whose height was adjusted as per the convenience of the participant. The eye tracker needs an initial calibration which is carried out using the SDK provided by the manufacturer. The goodness of calibration is represented on a scale of 1-5 where 5 represents best calibration (error < 0.5 degree). We ensured that all the participants obtained a score of 5 in the calibration phase. Before starting the actual test, a demo session consisting of 5 trials is conducted to make the participants comfortable with the setup. The task began with a black fixation cross appearing on a white background for 15 seconds. This period is treated as the baseline period. This was followed by the task for each version of dDSST.

### Experimental Protocol

We carried out the 3 different experimental sessions (experiment 1, 2 and 3) using the designed versions. Firstly, we validated the performance characteristics of dDSST against the standard pDSST task. For this, we used version 1 of the dDSST as it is the basic version having close resemblance to the pDSST. Fourteen participants performed pDSST and version 1 of dDSST twice (once in morning, and again in afternoon). The order of pDSST and dDSST version 1 was randomized among the participants.

Next, we performed the test-retest validation of all the three versions of dDSST over 4 different sessions. This was necessary to analyze the consistency of the test with sessions. Five participants (two females) performed the three versions of dDSST for four sessions (2-morning (M) sessions and 2-afternoon (A) sessions). As each of these versions are meant to capture different information, the presentation sequence of these versions to the participants is not randomized and the following sequence is maintained – version 1 administered first, followed by version 2 and 3. Two morning sessions are denoted by time *t*1, *t*2 and two afternoon sessions are denoted by *t*3 and *t*4.

Finally, we explored the age-related effects on the overall performance. In order to do so, eye tracking and gaze behavior of the participants recorded by the eye tracker are analyzed for both *C1* and *C2* groups.

## Results and Discussions

As mentioned earlier, in our present research, we have carried out three Experiments. Here we present our observations for the three experiments.

### Experiment 1: Validation of dDSST with pDSST

In order to be satisfied with the experimental setup and the environment, we carried out a basic test-retest check using two different sessions (i.e., Morning session (A) and Afternoon session (A)). The Table 5 and Table 6 show the test performance metrics for the morning and afternoon sessions carried out on the 14 participants. It is to be noted that the difference between the performance metrics is not statistically significant (p>0.05 using Mann-Whitney’s U Test [31]) indicating that both the pDSST and dDSST version 1 passed the test-retest check in the current experimental setup.

**Table 5:** pDSST test performance metrics (Average±SD) for the morning (M) and afternoon (A) sessions. Note: The score for pDSST is out of 100.

Test	Total Time (seconds)	Score (for 100 trials)
pDSST (M)	162.714 (±26.004)	99.99 (±0.002)
pDSST (A)	148.5 (±17.553)	99.929 (±0.267)
*p*-value (effect size)	0.09 (±0.31)	1 (0)

**Table 6:** dDSST test performance metrics (Average ±SD) on 50 trials for the morning (M) and afternoon (A) sessions.

Test	Mean Response Time for each trial (Seconds)	Score (for 50 trials)
dDSST (M)	1.987 (±0.083)	49.642 (±0.633)
dDSST (A)	1.969 (±0.064)	49.571 (±0.513)
*p*-value (effect size)	0.53 (0.11)	0.56 (0.1)

For the validation of the designed digitized version (dDSST version 1) with respect to conventional pen and paper version, we calculated the Pearson’s correlation coefficients [32] on the time taken to complete both the versions. The results are presented in Table 7. We found a high correlation (*r*=0.76) between the dDSST related scores achieved by the participants during the morning and afternoon sessions. Also we carried out validation of dDSST against the pDSST and the correlation between the performance across M and A sessions are shown in Table 7.

**Table 7:** Validation of the dDSST basic version (version 1) against the pDSST using Pearson’s correlation *coefficient* with *p*-values given in brackets.

	**pDSST (M) & pDSST (A)**	**dDSST (M) & dDSST (A)**	**pDSST (M) & dDSST (M)**	**pDSST (A) & dDSST (A)**
Correlation coefficient	0.91 (<0.001)	0.76 (0.001)	0.51 (0.062)	0.48 (0.08)

### Experiment 2: Test-Retest validation of three versions of dDSST

**Figure 9.**
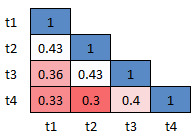
Inter-sessions test-retest validation for version 1: Heatmap showing the average p-values for the 4 sessions across participants

**Figure 10.**
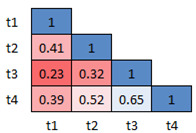
Inter-sessions test-retest validation for version 2: Heatmap showing the average p-values for the 4 sessions across participants

**Figure 11.**
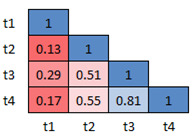
Inter-sessions test-retest validation for version 2: Heatmap showing the average p-values for the 4 sessions across participants

The mean *p*-values (computed using Mann-Whitney’s U Test) for all the three versions of dDSST across sessions are shown in Fig. 9, 10 and 11, respectively. The mean *p*-values for the response time of all the 5 participants for the Class D samples are calculated. The idea is to check whether there exists a significant difference in the data between morning and the afternoon sessions. The figures show the heatmap of the *p*-values between the sessions. Red color is used for the lowest and blue for the highest *p*-values. The results show that the sessions do not have any statistical difference (*p*>0.05) indicating the similarity between the sessions. The range (minimum, maximum) of the mean effect size values for version 1, 2 and 3, are found to be (-0.05, 0.17), (-0.06, 0.19) and (-0.03, 0.29) respectively. This ensures the test-retest reliability of the 3 versions of the designed dDSST.

### Experiment 3: Results for age-related effects

**Figure 12.**
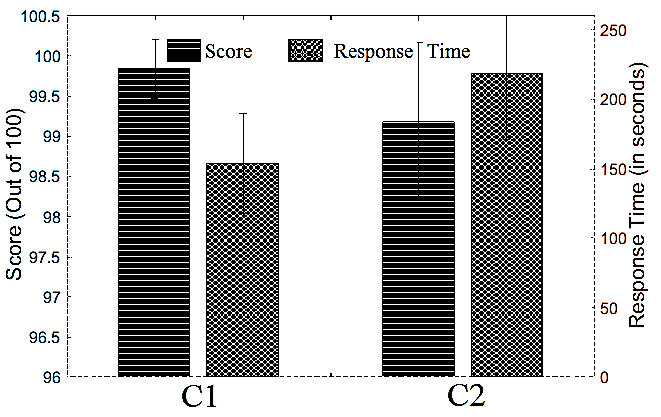
Average performance metrics for pDSST for the 2 age groups

Next we analyzed the performance of the participants belonging to two groups *C1* and *C2* respectively using the *UserResponse*, *Direct* features and the *Derived* features (equation 1). The Fig. 12 shows a comparative group analysis of the performance metrics e.g., score and response time for the pDSST task for both *C1* and *C2*. The participants in *C1* group scored slightly more (*p=*0.0395 using Mann-Whitney’s U test, effect size=0.4203) than participants in *C2* group in comparatively lesser total task completion time (*p* =0.0028, effect size=-0.6097). However, further exploration might be needed to infer the sources of these differentiated observations.

The Table 8 shows the performance (in terms of score, response time and Key hold duration) of both the age groups for all three versions of dDSST task. The results are averaged over all the participants. The average response time varied significantly (*p*<0.05, effect size = -0.4494, -0.5322 and -0.5677, respectively for the 3 versions of dDSST) for all the three versions of dDSST. Though there are minor differences in score and key hold time, these differences are not statistically significant (*p*>0.05). The effect sizes for key hold time are -0.047, -0.09 and -0.22 respectively for three versions of dDSST. Similarly, the effect sizes for score considering the three versions of dDSST are -0.1, 0.015 and -0.16, respectively. The user responses obtained from the digitized versions of DSST are not adequate to draw proper conclusions about the sources of differences. Thus, this necessitates use of additional measures like physiological sensing that can offer deeper insights into the user’s behavior while performing the task.

**Table 8:** Averaged performance (±SD) metric values for the 3 versions of the dDSST

	**Version 1**		**Version 2**		**Version 3**	
	**C1**	**C2**	**C1**	**C2**	**C1**	**C2**
**Score (out of 50)**	49.15 (0.8)	48.9 (1.92)	49.69 (0.63)	49.72 (0.46)	49.38 (0.65)	49.09 (1.9)
**Response Time (sec)**	1.104 (0.18)	1.29 (0.2)	1.154 (0.2)	1.35 (0.19)	1.16 (0.15)	1.37 (0.19)
**KeyHold Time (sec)**	0.137 (0.036)	0.137 (0.039)	0.126 (0.03)	0.133 (0.036)	0.123 (0.031)	0.138 (0.033)

**Figure 13.**
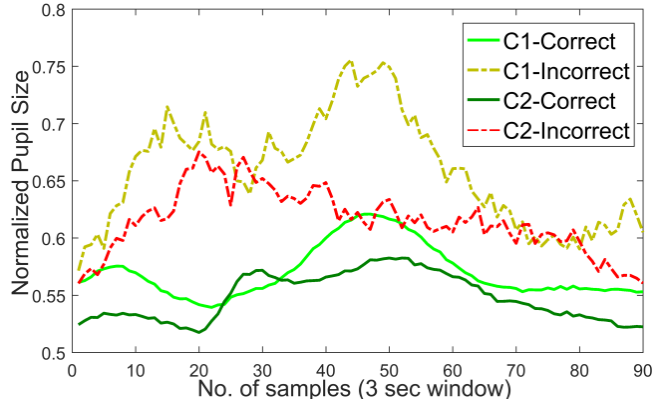
Average pupillary response for correct and incorrect responses for dDSST v1

Literature review indicates that often pupil size can be representative of one’s awareness or metacognitive confidence with which an individual is performing a task [33, 24]. Also, the effects of cognitive load can also be quantified by studying spontaneous pupillary responses [23]. Fig. 13 shows the normalized average variations in pupil size for the correct and incorrect responses made by the participants. The Eye Tribe eye tracker provides pupil dilation data in arbitrary units. We have performed the normalization for the sake of visual comparison. For this study we selected the data window length of 3 seconds from the user response as the duration of each trial is 3 seconds. The pupil size data across correct and incorrect responses are found to be statistically different (*p*<0.05, effect size=-0.7872), (*p*<0.05, effect size =-0.8057) for both *C1* and *C2* groups respectively. It can be seen from this figure that there is an increase in pupil size for incorrect responses that might be indicative of the awareness related to cognitive functioning. In other words, the participants might have a sense of correctness of their responses even in the absence of any feedback. This however needs further exploration with the stimulus designed particularly for the assessment of meta-cognitive awareness as given in [32]. The difference in the pupillary responses between the 2 groups (*C1* and *C2*) is statistically significant during both the correct and incorrect trials ( *p*<0.05, effect size = 0.3803 and 0.4436 for correct and incorrect trials, respectively).

**Figure 14.**
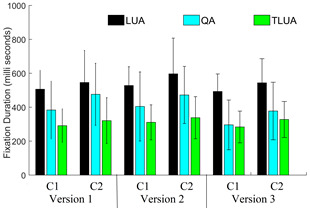
Average fixation durations in LUA, QA and TLUA for 3 different dDSST versions and 2 age groups

Fig. 14 shows our findings on average fixation duration in the lookup area (LUA), query area (QA) and the target LUA (TLUA). In version 1, all the 3 regions- LUA, QA and TLUA have equal role. In version 2, LUA and TLUA entries keep changing with the trials. The percentage increase in the average fixation duration for *C2* group with respect to *C1* group for the LUA and TLUA are 13 and 8.8, respectively. In version 3, the QA keeps changing with the trials. For version 3, the percentage change in the average fixation duration for QA is found to be 27.53. Again, compared to the *C1* group, the *C2* group of participants spent comparatively longer time in LUA and QA when compared to the *C1* group. Such an observation can be possibly attributed to the age related decline in cognitive processing required to match the two entities. Again participants belonging to *C1* group spent relatively less time in TLUA compared to QA, suggesting that they are able to memorize and recognize the query pair better in comparison to the *C2* group.

**Figure 15.**
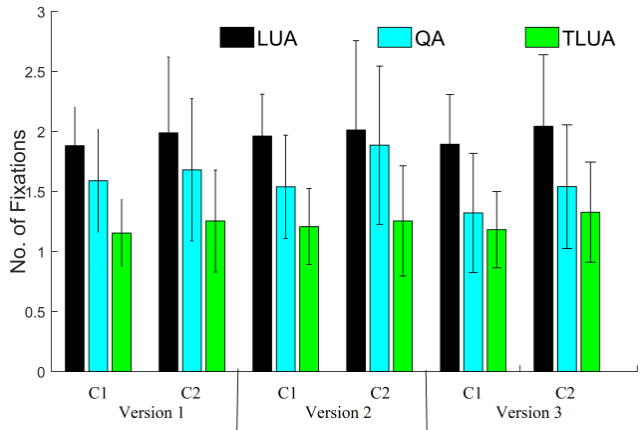
Average number of fixations in LUA, QA and TLUA for 3 different dDSST versions and 2 age groups

If an individual is not looking at the right location of the TLUA (as evident from the fixation gaze coordinates), then it might reflect that the individual is not able to understand the task properly. Another important observation is that, the fixation duration for younger participants (*C1* group) during both version 1 and version 2 are comparable. In contrast, older participants ( *C2* group) could not fixate properly during version 2 where the digit-symbol pair in TLUA is changing in each trial. A similar trend is also observed in the variations of number of fixations across participants as shown in Fig. 15. As mentioned earlier, for version 2, the LUA and TLUA have major roles to play. For the *C2* group, the percentage changes in the average number of fixations for LUA and TLUA with respect to the *C1* group are 2.55 and 3.93, respectively. Similarly, for version 3, QA plays a key role and the percentage difference in the average number of fixations in this case is 16.59 for both the age groups.

**Figure 16.**
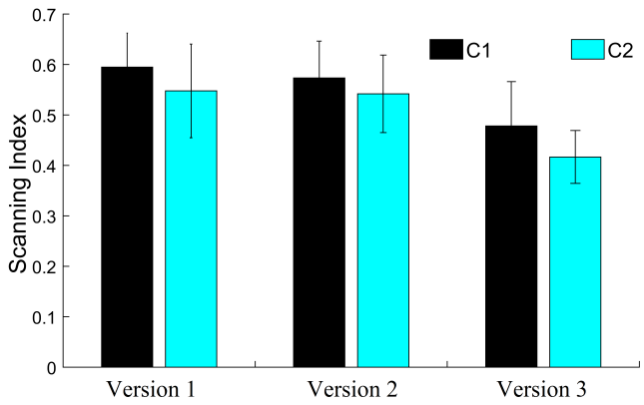
Scanning index for class D fixation data for 2 age groups

The results for Scanning index (*S*) given in equation 2 for all three versions of the dDSST are shown in Fig. 16. The percentage change in average *S* for the *C2* group with respect to the *C1* group is -7.94, -5.52 and -12.84, respectively for the three versions of the dDSST. However, we need to validate these results on participants with visual neglect and other cognitive impairments.

For dDSST Version-3, the results of Performance index ( *H*) (equation 3) corresponding to various gaze related features are given in Table 9. Since all of our participants are devoid of any cognitive impairment we did not notice much variation in the performance index. However, we found significant difference ( *p*<0.05) in Performance index computed for the number of fixations in the QA. This reflects that the metric is capable of capturing the difference in visuo-spatial characteristics between the two age groups and is found to be statistically significant. This is in accordance with the Performance index detecting the visuo-spatial [27] differences with respect to the QA that changes in each trial in the case of version 3 for the two age groups considered.

**Table 9:** Performance index (Average±(SD)) for 2 age groups (C1 and C2)

*H* computed on	**C1**	**C2**	***p*-value (effect size)**
**Pupil Size**	0.9998 (0.0001)	0.9981 (0.0058)	>0.05 (-0.0828)
**Score**	0.9996 (0.0004)	0.9992 (0.0018)	>0.05 (-0.1461)
**LUA nFixs**	0.9844 (0.01)	0.9879 (0.0056)	>0.05 (-0.1183)
**QA nFixs**	0.9715 (0.043)	0.9887 (0.012)	<0.05 (-0.4376)

**Figure 17.**
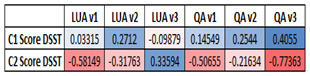
Correlations among the eye tracking variables in 3 versions of dDSST against the dDSST score for the two age groups

The correlations between the gaze related variables and the dDSST scores are also computed (heatmap in Fig. 17) to study the relationship between the user responses and the gaze-related features. We have not found any strong correlation between the two parameters for the *C1* group. In contrast, there is considerable negative correlation for the *C2* group. This is important since the participants belonging to the *C1* group tend to either remember the LUA entries or they spend less time in scanning the LUA and QA whereas the participants belonging to the C2 group tend to spend more time in scanning the LUA and QA. To summarize, gaze related features can be studied as markers for detecting age related variations in working memory and perception.

## Conclusions

In our present research we designed a digital version of DSST (dDSST) presented with different variations and validated against the standard pen and paper based DSST (pDSST). Also, we integrated an Eye Tracker to our setup to study the gaze-related variations corresponding to participants performing the dDSST. We performed three types of experiments namely, (i) Experiment 1: validation of the dDSST by comparing against the existing pDSST, (ii) Experiment 2: test-retest validation of different versions of dDSST and (iii) Experiment 3: analysis and evaluation of the age related differences in the user response, eye gaze behavior and pupillary characteristics. Our results show that the digitized version of the DSST can be a more reliable and a valid test to assess the cognitive functionality of an individual compared to the traditional pen and paper version. In addition to the test score and the task completion time, the digitized version provides trial wise information and eases the way of including physiological sensing. The pen and paper version can assess the performance of an individual in terms of number of correct entries. However, the digitized version captures relatively finer details like possible underlying reasons behind the performance of user with the help of physiological sensing. The digital version can also be used for studying age related differences in test performance. Results show that it is possible to derive useful information from features like gaze duration, number of fixations of participants in the specific regions of interest and so on. Our proposed system can be used in periodic screening like the one used in rehabilitation applications. The results confirm the potential of our designed digital version to be used as an early marker of cognitive dysfunction and problem related to working memory. Finally, our digital version can be used for assessment as well as for practice sessions, thereby serving as a complementary tool in the hands of skilled professionals.

This is still effectively a “proof of concept” study and any patterns that are commented on need to be treated as indicative. Further work is required in the direction of targeting participant groups with various cognitive dysfunctions. We also have plans to test all the dDSST versions on participants of various other age groups.

## Ethics and Conflict of Interest

The author(s) declare(s) that the contents of the article are in agreement with the ethics described in http://biblio.unibe.ch/portale/elibrary/BOP/jemr/ethics.html.

Authors Debatri Chatterjee, Rahul Dasharath Gavas, Kingshuk Chakravarty, Aniruddha Sinha are employed as researchers by TCS Research and Innovation, Tata Consultancy Services Ltd., India. Author Dr. Uttama Lahiri is Associate Professor of Electrical Engineering at IIT Gandhinagar, India. Additionally, she is one of the core team members of Cognitive Science at IIT Gandhinagar. She has received honorarium as a research advisor from TCS Research and Innovation, Tata Consultancy Services Ltd. during the tenure of this work. All the authors declare no other competing interest.

## Acknowledgements

We would like to thank all the participants for their valuable time and cooperation throughout the experiment. We also thank Venkatasubramanian Viraraghavan for his valuable inputs for sensor signal processing approaches.
